# *RNF114* Silencing Inhibits the Proliferation and Metastasis of Gastric Cancer

**DOI:** 10.7150/jca.62033

**Published:** 2022-01-01

**Authors:** Zongfeng Feng, Leyan Li, Qingwen Zeng, Yang Zhang, Yi Tu, Wenzheng Chen, Xufeng Shu, Ahao Wu, Jianbo Xiong, Yi Cao, Zhengrong Li

**Affiliations:** 1 Department of General Surgery, the First Affiliated Hospital of Nanchang University, Nanchang, China.; 2 Laboratory of Digestive Surgery, Nanchang University, Nanchang, China.; 3 Queen Mary School, Medical Department of Nanchang University, Nanchang, China.; 4 Department of Pathology, the First Affiliated Hospital of Nanchang University, Nanchang, China.

**Keywords:** *RNF114*, EGR1, miR-218-5p, methylation, ubiquitylation, gastric cancer

## Abstract

RNF114 (E3 ubiquitin ligase RING finger protein 114) was first identified as a zinc-binding protein that promotes psoriasis development; however, its role in gastric cancer is still unclear. We explored the relationship between *RNF114* and gastric cancer using bioinformatics and molecular biology techniques. The results showed that RNF114 was highly expressed in gastric cancer and negatively correlated with the patient's prognosis. Functional assays suggested that *RNF114* silencing suppressed the proliferation and metastasis of gastric cancer cells to a certain extent. Further studies showed that* RNF114* expression was potentially targeted by miR-218-5p and methylation modification, and mediated downstream EGR1 (early growth response 1) degradation by the ubiquitylation approach. Together, the present results highlight the detrimental effects of *RNF114* overexpression in gastric cancer and contribute to a better understanding of the mechanisms underlying *RNF114* functionality.

## Introduction

In 2020, gastric cancer (GC) was the fifth most commonly diagnosed malignant tumor and the third leading cause of death in numerous countries, especially the coastal countries of Southeast Asia, with more than 1 million newly diagnosed cases and an estimated 783,000 deaths 1. Although surgical and chemotherapy treatments have constantly improved, the survival rate of patients with GC remains relatively low 2,3. Therefore, it is necessary to uncover the epigenetic mechanism of GC occurrence and development.

RNF114, also known as ZNF228 or ZNF313, is a member of the zinc-binding protein family 4,5. The *RNF114* gene is located on chromosome 20q13.13, which is recognized as a common gene amplification region 6. *RNF114* encodes a protein containing both C2H2 and RING finger zinc-binding domains, which binds to ubiquitin via the C-terminal ubiquitin interaction motif (UIM) and exerts E3 ligase activity through the N-terminal RING domain 7. The RNF114 protein is expressed widely in many normal tissues, such as the heart, liver, and kidney; however, it is decreased in the muscle, lung, and placenta 8. The RNF114 protein is a novel determinant of psoriasis susceptibility, and its overexpression is a crucial indicator of epithelial inflammation 9-11. Boren *et al*. 12 reported that RNF114 mediates osteoclastogenesis via the RANKL/RANK/TRAF6 signaling pathway. Huang *et al*. 13 demonstrated that Ubqln4 negatively interacts with RNF114 to inhibit the proliferation of tumor cells. Gopeshwar *et al*. 14 identified that *RNF114* is upregulated by DNA copy number increases and is a potential oncogene in cervical cancer. There are an increasing number of reports regarding RNF114 in tumors, whereas detailed reports regarding its role in GC are lacking.

EGR1, a transcription factor, plays a dual role in tumor progression by regulating downstream target genes. EGR1 may suppress tumor effects in breast tumors 15, rhabdomyosarcoma 16 and bladder tumors 17, while promoting tumor effects in prostate cancer 18 and lung cancer 19. Surprisingly, contrary results have been reported for the role of EGR1 in gastric cancer 20,21, the mechanism of which is still unclear. MicroRNAs (miRNAs), 22 nucleotides on average, are a group of evolutionarily conserved single-stranded non-coding RNAs. miR-218-5p has been implicated in various types of cancer 22. As a tumor suppressor, miRNAs have been found to be downregulated in GC. Tianqi suggested that the long non-coding RNA SNHG12 promotes GC metastasis by regulating the miR-218-5p/YWHAZ axis 23, while Min found that miR-218 suppresses gastric cancer cell cycle progression through the CDK6/Cyclin D1/E2F1 axis 24.

In this study, we analyzed the characteristics of variant *RNF114* mRNA levels in patients with GC. These results demonstrated that *RNF114* was highly expressed at the mRNA or protein level in GC, and that patient prognosis was more reliant on upregulated RNF114 than downregulated levels. In addition, miR-218-5p is a potential regulator that controls *RNF114* expression, to some extent, and can mediate EGR1 degradation via the ubiquitylation approach.

## Materials and methods

### Bioinformatic analysis

The differential expression of RNF114 across various cancers was investigated using TIMER (https://cistrome.shinyapps.io/timer/) 25, GEPIA (Gene Expression Profiling Analysis) (http://gepia.cancer-pku.cn/) 26, and Oncomine databases (https://www.oncomine.org/) were used to analyze RNF114 expression in GC. RNA-seq expression profiles of GC were downloaded from The Cancer Gneome Atlas (TCGA) database (https://portal.gdc.cancer.gov/) and processed using R software. These files were composed of sample expression files and phenotypic classification files (Format: .gct and .cls). We analyzed these datasets for signaling pathways of the *RNF114* gene using GSEA software 4.0.3. The correlation between *RNF114* mRNA expression and overall survival (OS) in GC was analyzed using the Kaplan-Meier plotter (http://kmplot.com/) 27, which is the most comprehensive survival website. DNA methylation of RNF114 was analyzed using MethSurv (https://biit.cs.ut.ee/methsurv/) 28, which is an interactive web portal that provides survival analysis based on DNA methylation biomarkers using TCGA data. Potential miRNAs and transcription factors associated with RNF114 were predicted using NetworkAnalysis3.0 (https://www.networkanalyst.ca/) 29, a comprehensive network visual analytics platform for gene expression analysis. Potential miRNAs were obtained and the co-expression relationship between potential miRNAs and targeted genes was analyzed using the starBase v2.0 (http://starbase.sysu.edu.cn/) 30, which mainly focused on miRNA-target interactions.

### Patient data and tissue samples

We collected 19 fresh GC tissues and peritumoral normal gastric mucosa at the Department of General Gastrointestinal Surgery of the First Affiliated Hospital of Nanchang University in 2018. Paraffin-embedded GC samples (n=123) and normal gastric gland samples (n=20) were obtained from the Department of Pathology of the First Affiliated Hospital of Nanchang University.

### Quantitative real time PCR (qRT-PCR) and western blotting

The qRT-PCR experiments were performed according to the manufacturer's protocol and according to a previous study 31,32. The *HACTB* gene was used as an endogenous control. HACTB: forward 5′-CATCCGCAAAGACCTGTACG-3′; reverse 5′-CCTGCTTGCTGATCCACATC-3′, RNF114: forward 5′-GGGAGACCCCAACTACCG-3′; reverse 5′-AGCGCTGCAACACCTGAT-3′. Western blotting was performed according to the manufacturer's protocol, as described in a previous study 33. The following antibodies were used in this study: β-actin (1:1000 dilution, Affinity, China) and RNF114 (1:700 dilution, Altas, Sweden).

### Immunohistochemistry (IHC) and evaluation

Paraffin-embedded samples were sliced into 3.5 µm thick sections, which were coated with rabbit anti-RNF114 antibody (1:200 dilution) after deparaffinization, rehydration, and antigen repair. Two independent pathologists evaluated the stained slides using a microscope (Nikon, Tokyo, Japan). Each case was scored based on the following criteria: intensity (0, negative; 1, weak (+); 2, moderate (++); 3, strong (+++)) and percentage of positive staining, area (0: 1-25%; 2: 26-50%; 3: 51-75%; 4: 76-100%). The ultimate score was the product of the intensity and area (0-12). Samples with a score ≥6 were defined as having high RNF114 expression, while samples with a score <6 were regarded as having low RNF114 expression.

### Cell culture, infection, and siRNAs

All GC cell lines, including HGC-27, MKN-45, MGC-803, SGC-7901, BGC-823, AGS, and the normal gastric epithelial cell line (GES-1), were maintained in RPMI 1640 medium with 10% fetal bovine serum (HyClone GE Healthcare Life Sciences, Logan, UT, USA) at 37 °C in a 5% CO_2_ atmosphere.

We obtained three sequences of RNF114 small interfering RNA (Si-1: sense 5′-GCCACCAUUAAGGAUGCAUTT-3′, antisense 5′-AUGCAUCCUUAAUGGUGGCTT-3′ Si-2, sense 5′-GUGGAACACUGCAAAUUAUTT-3′, antisense 5′- AUAAUUUGCAGUGUUCCACTT-3′; Si-3: sense 5′-GUGGCUACUUGUUCCAAAUTT-3′, antisense 5′-AUUUGGAACAAGUAGCCACTT-3′) and a negative control RNA from GenePharma Company (Shanghai, China). These siRNAs and negative control RNA were transfected into HGC-27 and BGC-823 cells.

The lentivirus vector included a short hairpin RNA of knockdown RNF114 expression (shRNA) and a negative control sequence (shNC) designed by GenePharma Company. We used shRNF114 (5′-GTGGCTACTTGTTCCAAAT-3′) and shNC (5′-TTCTCCGAACGTGTCACGT-3′) to infect HGC-27 and BGC-823 cells with polybrene (5 μg/mL). We tested transfection efficiency and selected using puromycin for subsequent cell functional experiments.

### Immunofluorescence (IF)

Tissue IF and cell IF were performed as described previously 34. Primary antibodies against RNF114 (1:100 dilution) were used in this study. Samples stained for RNF114 were incubated with an Alexa Fluor 594-conjugated goat anti-rabbit secondary antibody (Elabscience, China), and the nuclear samples were stained with DAPI.

### Cell function assays

#### 5-Ethynyl-2′-deoxyuridine (EdU) assay

Lentivirus-transfected GC cells were cultured in 96-well plates. First, 200 µL of a 50 µmol/L EdU solution (Guangzhou RiboBio Co.,LTD, China) was added to the cultured cells. After washing, fixation, and permeabilization, 100 µL of 1× Apollo solution was added to the cells. Cellular DNA was then stained with 100 µL of 1× Hoechst 33342 solution. A fluorescence microscope was used to capture images of EdU- and Hoechst 33342-positive cells.

#### Colony formation

The cells were seeded in 6-well plates and cultured at 37 °C in a 5% CO_2_ atmosphere for 10-14 days. Colonies were fixed with 4% paraformaldehyde, stained with 0.5% crystal violet solution, and washed before counting.

#### Transwell assay

Transwell chambers with 8 µm pores with or without Matrigel (BD Biosciences) were used for invasion and migration assays, as described in a previous study 35. The chamber was fixed with paraformaldehyde, stained with crystal violet, and observed under a microscope.

#### Scratch-wound assay

The cells were then placed in sterile 6-well plates. A “wound” was scratched using a sterile 10 µL pipette tip when cells were grown to 80-100% confluence. Images were obtained using a microscope at 0 and 24 h, and analyzed.

### Methylation-specific PCR (MSP)

Genomic DNA was isolated from cells samples by using columnar centrifugation, and was treated with sodium bisulfite. PCR was performed using methylated and unmethylated primers, respectively. methylated primers: forward 5′- AGTGTAAGTGGTGTCGAATTAACGAT -3′; reverse 5′- AAACTAACGAATAAACCGCCCCT-3′, unmethylated primers: forward 5′- AGTGTAAGTGGTGTTGAATTAATGATAAA -3′; reverse 5′- CAAAACTAACAAATAAACCACCCCTC -3′. PCR products were identified with agarose gel electrophoresis.

### *In vivo* tumor xenograft model

Ten mice were purchased from SJA Laboratory Animal Co., Ltd (Hunan, China) and randomly separated into shNC and shRNA groups. A total of 1 × 10^6^ cultured shNC-BGC-823 and shRNF114-BGC-823 cells were diluted with PBS and injected into the right thigh of each nude mouse. After 1 week, subcutaneous xenograft tumors were observed, and the tumor size and body weight were measured every week. Five weeks later, the tumors in the right thigh of nude mice were resected subtly after sacrifice, and their weights and sizes were measured. All procedures in this study were approved by the Institutional Animal Care Committee of the First Affiliated Hospital of Nanchang University.

### Statistical analysis

Data were processed using SPSS version 19.0 (SPSS, Chicago, USA) and GraphPad Prism 7.0 software (GraphPad Software, La Jolla, CA, USA). Student's *t-*test or one-way analysis of variance was used to analyze differences between groups. The chi-square test and Fisher's exact test were used to compare the relationship between RNF114 expression and clinicopathology. Kaplan-Meier curves and Cox regression models were used to analyze survival rates. All data are expressed as means ± SD, and *P* values <0.05 were considered statistically significant.

## Results

### RNF114 overexpression was observed in GC using bioinformatics analysis

**Figure 1:**
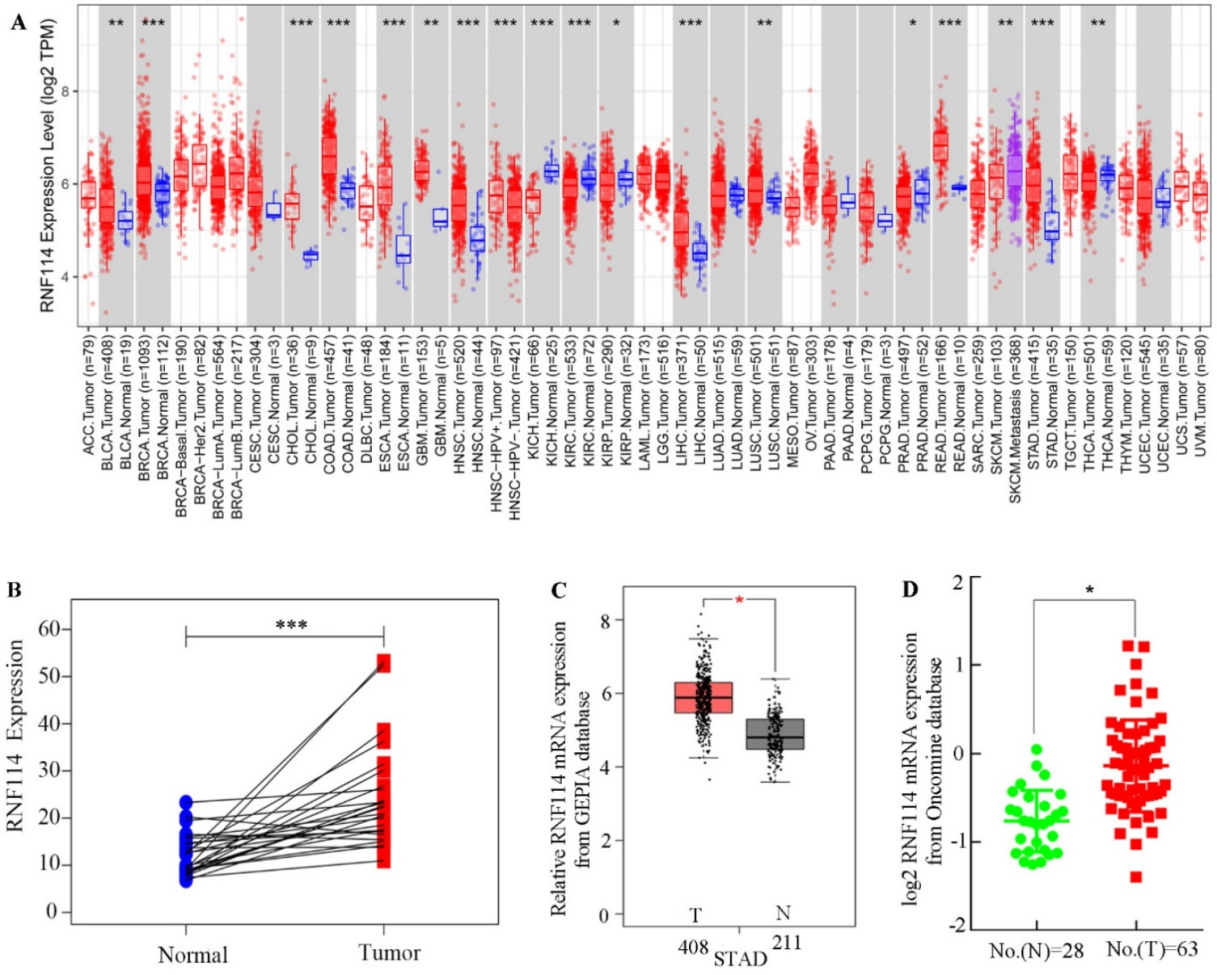
** RNF114 was overexpressed in gastric cancer. (A)** The different expressions of *RNF114* mRNA among pan-cancer from TIMER database. **(B)** Differences in *RNF114* expression for GC were analyzed using a paired *t*-test analysis. **(C)** RNF114 was overexpressed in GC according to the GEPIA database. **(D)** RNF114 was overexpressed in GC with Chen sample data according to the Oncomine database (***p≤0.001, **p≤0.01, *p≤0.05).

To explore *RNF114* mRNA expression among different tumors, TCGA datasets associated with tumors were extracted and calculated using the TIMER server. As shown in Figure 1A, *RNF114* mRNA was notably overexpressed in most lethal tumors, particularly in GC. Meanwhile, pair-differed analysis indicated that RNF114 expression was markedly higher in the tumor than in the normal group (Figure 1B). Combined with the GTEx and TCGA GC datasets, increasing the normal sample, *RNF114* mRNA expression was highly expressed in GC compared with normal samples (Figure 1C). Chen data, extracted from the Oncomine database, revealed similar outcomes regarding *RNF114* expression (Figure 1D). The bioinformatics results suggested that *RNF114* mRNA was overexpressed in GC, and this finding requires further study.

### RNF114 was elevated in GC tissues and poor outcome was observed with high RNF114-expression patients

**Figure 2:**
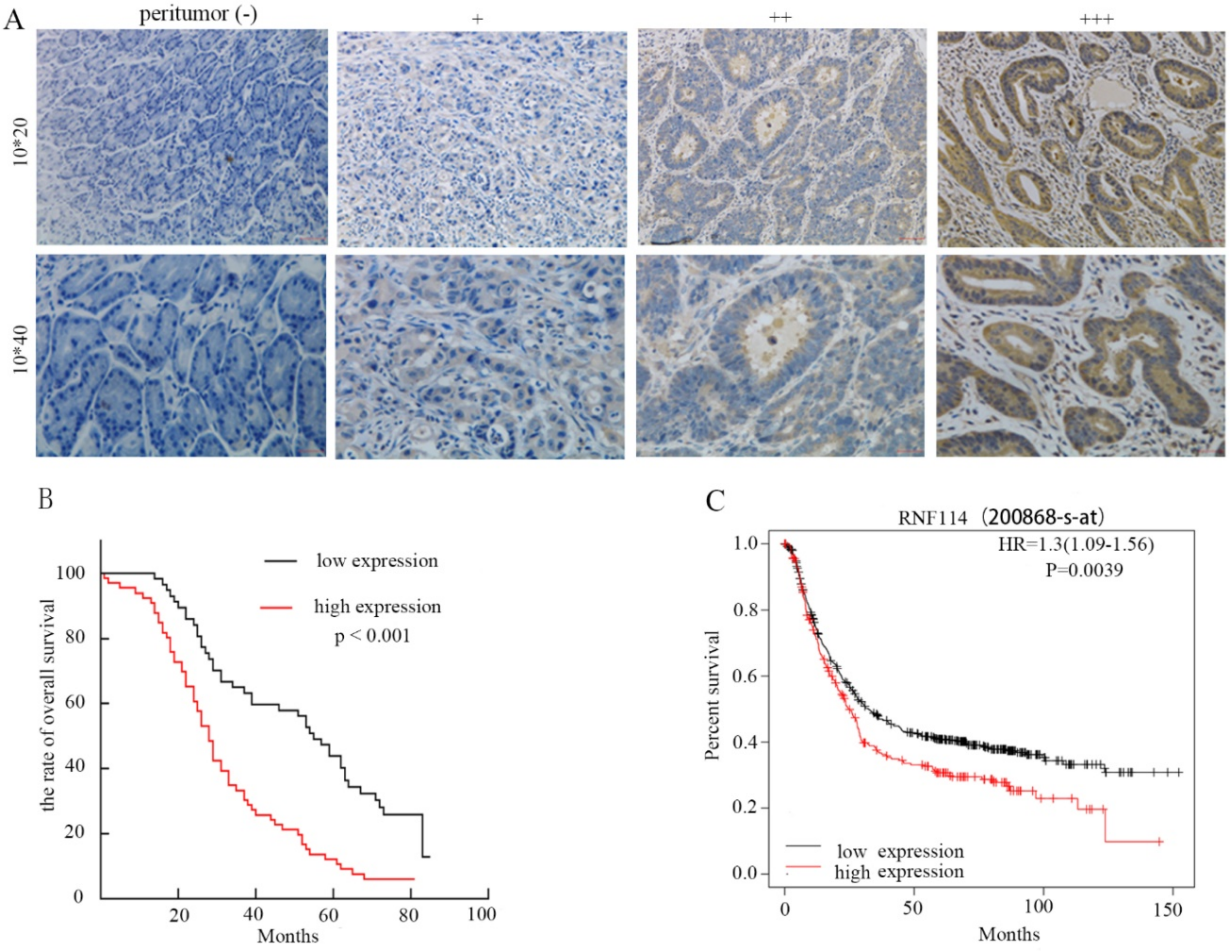
** Analysis of survival with GC based on RNF114 expression via IHC. (A)** Representative images of RNF114 staining in GC and adjacent normal tissues (+: weak, ++: moderate, +++: strong, 10*20 and 10*40 respectively represents an image magnified 200 times and 400 times). **(B)** Kaplan-Meier overall survival analysis of 123 patients stratified by RNF114 high and low expression levels. **(C)** The Kaplan-Meier Plotter database also showed that high expression of RNF114 correlated with shorter OS.

To investigate the correlation between RNF114 expression and clinicopathological characteristics in GC patients, we performed immunohistochemistry (IHC) and evaluated 20 normal gastric tissues and 123 primary GC tissues. The IHC results revealed that RNF114 expression was higher in tumor tissues than in adjacent normal tissues (Figure 2A). According to whether the coloring score of RNF114 protein was greater than or less than 6 points, all patients were divided into high expression and low expression groups. As described in Table 1, RNF114 protein expression was closely associated with tumor localization (*p*<0.001), T stage (*p=*0.003), and lymph node metastasis (*p*=0.001); however, RNF114 expression was independent of other parameters, including age, gender, tumor diameter, tumor differentiation, cancer nodule number, and vascular invasion. Compared with the low expression group, the high expression group had a worse overall survival (OS) (*p*<0.001), observed using Kaplan-Meier analysis (Figure 2B), which was also confirmed by the K-M Plotter database of Affymetrix gene chip ID: 200868_s_at (Figure 2C). Univariate and multivariate analyses showed that RNF114 levels (HR=1.772, *p*=0.015) were independent prognostic factors in patients with GC (Table 2).

**Figure 3:**
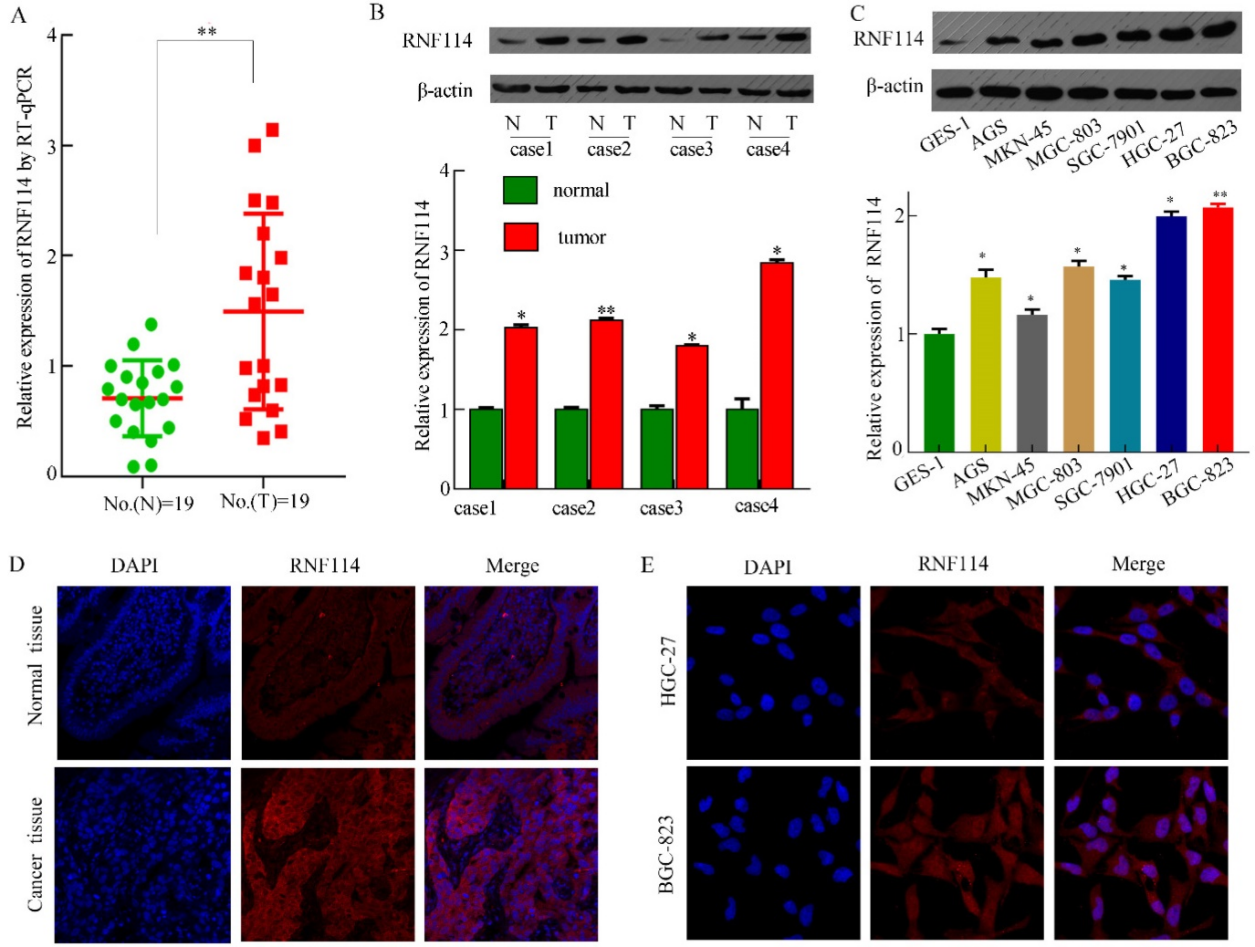
*** RNF114* was overexpressed in GC tissues and cells. (A-C)** RNF114 expression levels were high in GC tissues, as observed using qRT-PCR and western blotting. A indicates qRT-PCR results with fresh tissues, B indicates western blot results with fresh tissues, and C indicates western blotting results with cells. **(D-E)** The expression and localization of RNF114 were examined using immunofluorescence staining in tissues (Figure D) and BGC-823 cells (Figure E) (***p≤0.001, **p≤0.01, *p≤0.05).

**Figure 4:**
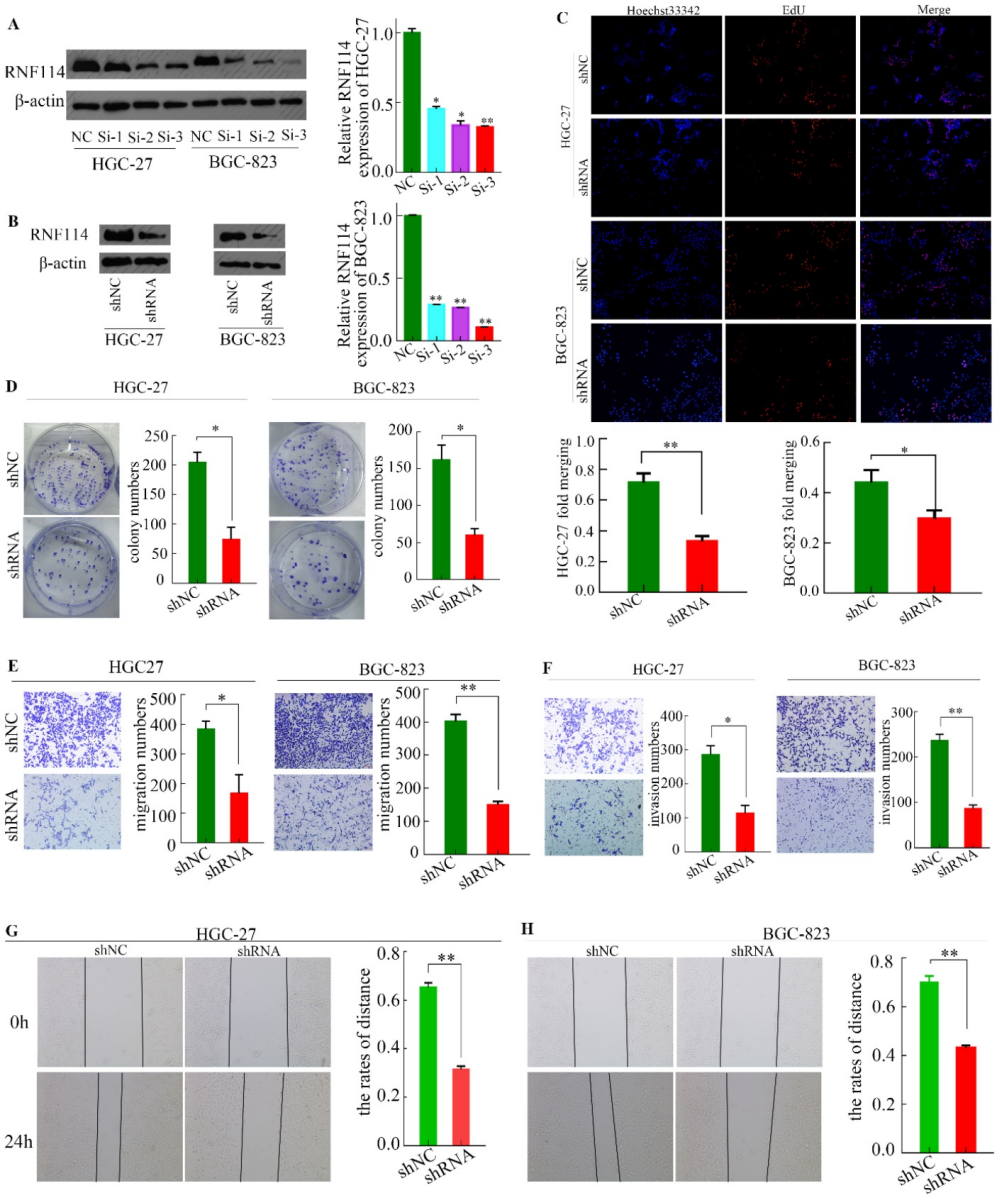
***RNF114* knockdown inhibits GC cell proliferation and invasion. (A)** The expression of RNF114 was downregulated by siRNA in HGC-27 and BGC-823 cells. **(B)** The expression of RNF114 was downregulated by shRNA in HGC-27 and BGC-823 cells. **(C)** The proliferation of cells with *RNF114* knockdown was analyzed using EdU assays. **(D)** Colony formation assays were used to detect the effect of RNF114 on cell growth. **(E-F)** Migration and invasion abilities of RNF114 knockdown cells were investigated using Transwell assays. E) Migration assays, F) invasion assays, and **(G-H)** scratch-wound assays were used to assess healing ability inHGC-27 cells (G) and BGC-823 cells (H) (***p≤0.001, **p≤0.01, *p≤0.05).

To assess *RNF114* expression in tissues and cells from RNA sequences and proteins, we detected *RNF114* mRNA and protein levels in 19 pairs of fresh GC tissues, contiguous normal tissues, and GC cells. As shown in Figure 3A, higher mRNA levels of *RNF114* were observed in GC tissues using qRT-PCR. Figure 3B shows a representative western blotting result from 19 pairs of fresh samples, and the expression of RNF114 protein in GC tissues was higher than that in the adjacent tissues. As shown in Figure 4C, RNF114 levels were lower in GES-1 cell lines than in GC cell lines, as determined by western blotting. Additionally, HGC-27 and BGC-823 cells showed higher expression than other cells. Subsequently, we performed IF staining of the GC tissues and cells. DAPI was used to stain the cellular nuclei (blue fluorescence), and the RNF114 protein was stained with a fluorescent antibody (red fluorescence). Red fluorescence (RNF114 protein staining) levels were lower in normal tissues than in cancer tissues (Figure 3D). In the two cell lines, RNF114 was mainly located in the cytoplasm and nuclei (Figure 3E). Based on the investigation of *RNF114*, it was elevated in GC tissues and poor outcome with high RNF114-expression patients.

### *RNF114* silencing decreased GC cell line proliferation, migration, and invasion

We designed three siRNAs (Si-1, Si-2, and Si-3) targeting RNF114 and an NC sequence to interfere with the expression of RNF114 in HGC-27 and BGC-823 cell lines. As shown in Figure 4A, all siRNAs exerted interference effects, with siRNA 3 showing the best effect. Thus, we selected Si-3 siRNA sequence for lentivirus packaging and subsequent infection of the two cell lines to constructe stable *RNF114*-knockdowned cells (Figure 4B).

EdU and colony formation experiments were performed to detect the proliferation ability of cells infected with the lentivirus. As shown in Figure 4C, the nuclei were stained with Hoechst 33342 (blue fluorescence), and the amplified DNA was stained with Apollo solution (red fluorescence). Using “ImageJ” software to scan the EdU-associated images, we found that approximately 36.95% of cells were positive for EdU in the shRNA group of HGC-27 cells, while approximately 77.42% was seen in the shNC group. Similarly, in the BGC-823 cell line, the proportion was approximately 42.21% in the shNC group and 29.73% in the shRNA group. Immediately after, the colony formation experiment showed similar trends (Figure 4D). To some extent, the cell proliferation ability of GC was weakened by *RNF114* silencing.

To identify the effect of *RNF114* on GC migration and invasion, we performed transwell and scratch-wound assays. The total number of migrated cells was lower in the shRNA group than in the shNC group (Figure 4E). The invasion ability was greater in the shNC group than in the shRNA group (Figure 4F). The control group healed faster in the scratch-wound assay than the *RNF114*-knockdown group for both HGC-27 and BGC-823 cell lines (Figure 4G-H).

### Signaling pathway with GSEA and the regulation network of *RNF114*

**Figure 5:**
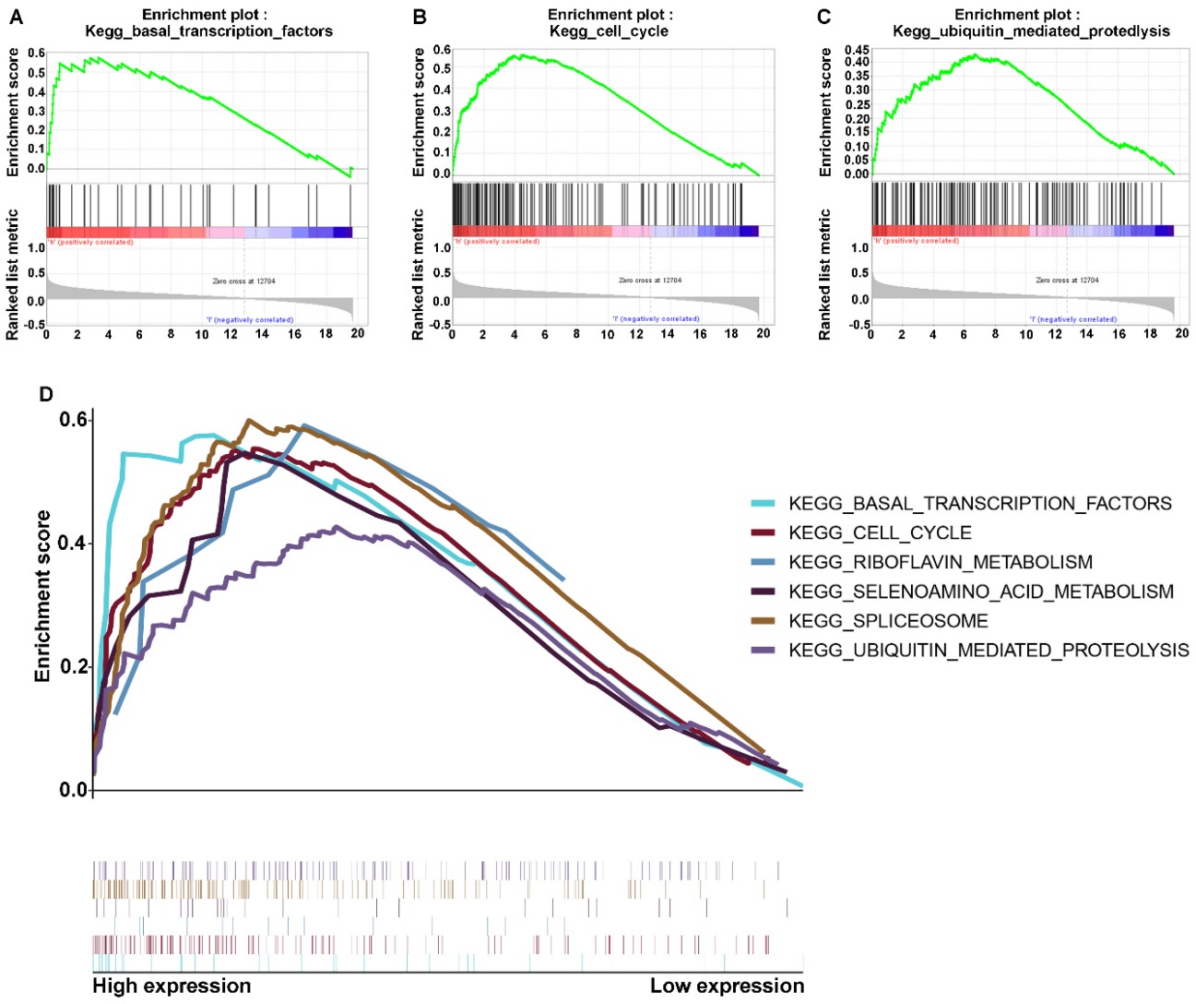
** Enriched signaling pathways associated with *RNF114* via GSEA. (A-C)** The signaling pathway genes were enriched in *RNF114* high expression. A indicates the ubiquitin-mediated proteolysis signaling pathway. B indicates the basal transcription factors signaling pathway. C indicates the cell cycle signaling pathway. **(D)** Multiple signaling pathways enriched in high *RNF114* gene expression.

The above results indicated that *RNF114* may be associated with proliferation and metastasis in GC, but its detailed regulatory mechanism remains elusive. To explore the involvement of *RNF114* in the signaling pathway, we assessed the co-expression between differentially associated gene expression with signaling and in two subtypes, which were divided by the expression of RNF114. We performed gene set enrichment analysis (GSEA), and the results showed that the malignant hallmarks of tumors, including ubiquitin-mediated proteolysis (normalized enrichment score [NES]=1.62, *p<*0.031), basal transcription factors (NES=1.81, *p<*0.001), and cell cycle signaling (NES=1.85, *p=*0.017), were dynamically correlated with the high RNF114 subtype (Figure 5A-C). In addition, the significant pathways with RNF114 expression in GC data also included spliceosome, selenoamino acid metabolism, and riboflavin metabolism, as shown in Figure 5D. Hence, binding RNF114 structure attributes, the ubiquitin-mediated proteolysis-signaling pathway may be implicated in the regulation of pivotal factors.

**Figure 6:**
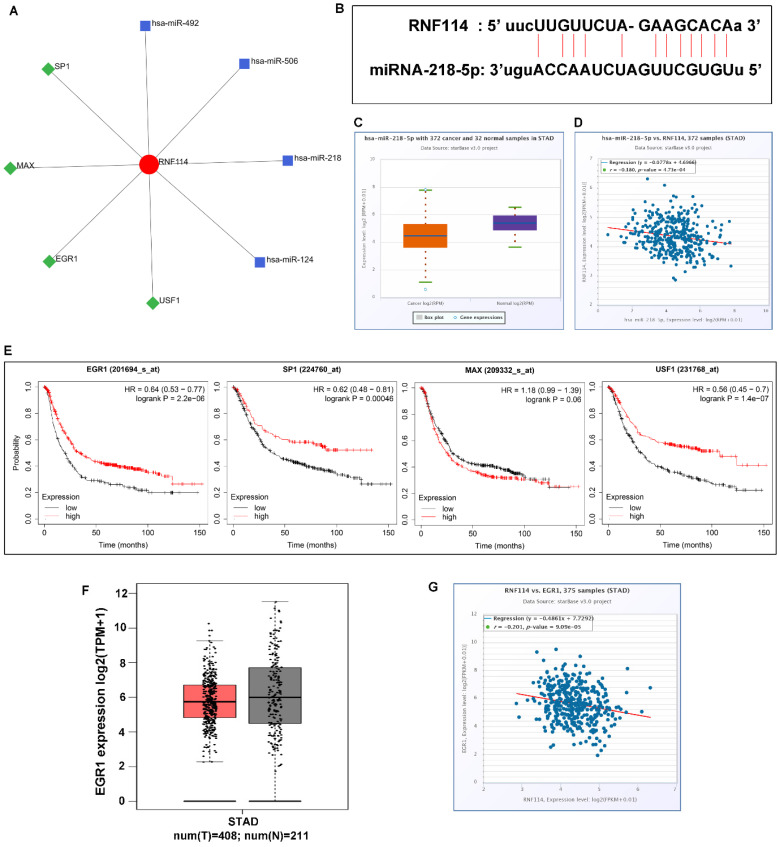
** Potential miRNA and transcription factor co-expression network in GC. (A)** miRNAs and transcription factors that interacted with RNF114 predicted by the NetworkAnalyst3.0 **(B)** The potential sequences of miR-218-5p binding sites at the 3′ UTR of *RNF114.* **(C)** miR-218-5p differential expression in GC and normal tissue from the starBase database. **(D)** Negative correlation between miR-218-5p and *RNF114* expression. **(E)** Result of EGR1, SP1, MAX, and USF1 prognostic analysis for GC in the Kaplan-Meier plotter databases. **(F)** EGR1 differential expression in GC and normal tissue from the GEPIA database (red represent tumor, grey represent normal). **(G)** The negative correlation between miR-218-5p and RNF114 expression from the starBase database.

To determine the molecular mechanism of *RNF114*, we analyzed the network of transcription factors and microRNAs using NetworkAnalyst3.0, a comprehensive network visual analytics platform for gene expression analysis (Figure 6A). There were four transcription factors and microRNA closely correlated with *RNF114*, respectively, which were SP1, MAX, EGR1, and USF1, and hsa-miR-492, hsa-miR-506, hsa-miR-218, and hsa-miR-124. Using the starBase databases, we found that hsa-miR-218-5p was associated with *RNF14*, the presence of an RNA-binding site is displayed in Figure 6B, and the differing and corresponding expression of hsa-miR-218-5p was evident in GC (Figure 6C-D). Down-expression of hsa-miR-218-5p may attenuate regulation and release RNF114 expression in GC. The association between transcription factors related to RNF114 and patient outcomes was analyzed using the Kaplan-Meier plotter database. The low expression of SP1, EGR1, and USF1 was associated with poor overall survival, while MAX expression was not significantly associated with the overall survival of patients with GC (Figure 6E). The trend of lower EGR1 expression in GC than in normal GC was analyzed using GEPIA from TCGA and GTEx databases (Figure 6F). Additionally, EGR1 expression was negatively correlated with RNF114, as concluded from the starBase database (Figure 6G). Based on these findings, we suggest that RNF114 may trigger the ubiquitylation degradation of EGR1 and indirectly contribute to GC development.

### *RNF114* methylation in GC

**Figure 7:**
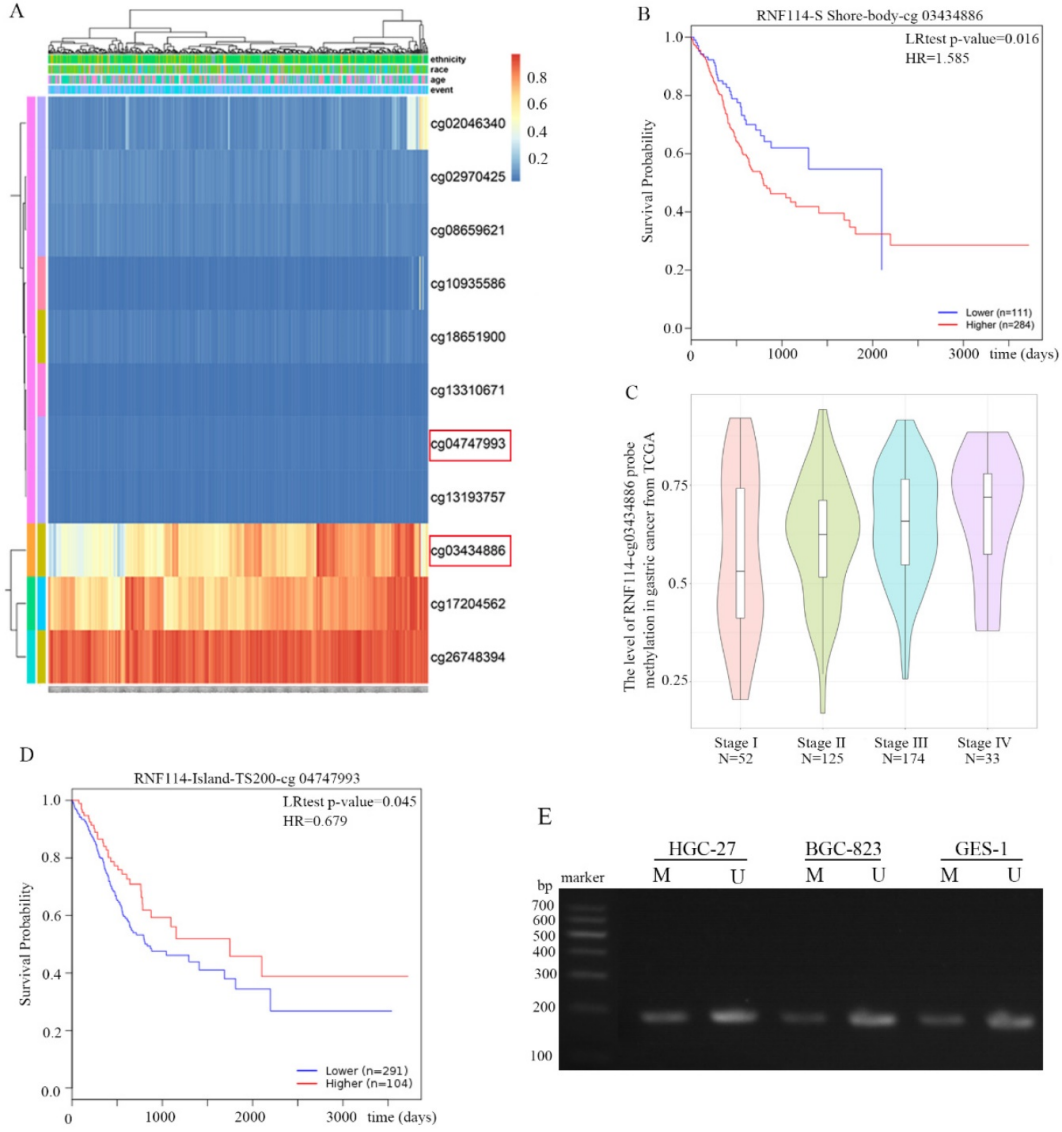
** DNA methylation of *RNF114* in GC from MethSurv network tool. (A)** The cluster of *RNF114* methylation in MethSurv network tool. Red to blue: hypermethylation to hypoexpression. **(B)** The Prognostic Values of methylation-associated probes cg03434886 with *RNF114*. **(C)** The relationship between RNF114-associated probe cg03434886 and tumor stage. **(D)** The Prognostic Values of methylation-associated probes cg4747933 with *RNF114*. **(E)** MSP detected the *RNF114* methylation in gastric cancer cells (M and U present methylated and unmethylated respectively).

DNA methylation of *RNF114* was analyzed using the MethSurv network tool from the GC TCGA database. All methylation probes associated with *RNF114* are shown in Figure 7A. The red and blue heatmaps represent hypermethylation and hypomethylation, respectively, in GC samples. Cg03434886 probes detected the *RNF114* gene in the hypermethylation region, and hypermethylation had a worse outcome than hypomethylation (Figure 7B). Further analysis revealed that the level of methylation was significantly associated with tumor staging, and the later the tumor staging, the higher the methylation (Figure 7C). This result revealed that *RNF114*-cg03434886 hypermethylation may stimulate *RNF114* expression in GC. However, *RNF114*-cg04747993 was less modified with methyl in GC chip results and the GC patient with *RNF114*-cg04747933 hypermethylation has a better prognosis than hypomethylation (Figure 7D). The methylation status of *RNF114* in the HGC-27, BGC-823 and GES-1 cell lines was detected using MSP. The bands in Figure 7E indicated *RNF114* partially methylated state, while more unmethylated *RNF114* in gastric cells. In general, gene methylation will lead to the inhibition of gene expression, nonetheless, *RNF114* has a controversial result, which is a very interesting phenomenon worthy of further study.

### *RNF114* silencing suppressed xenograft tumor growth

**Figure 8:**
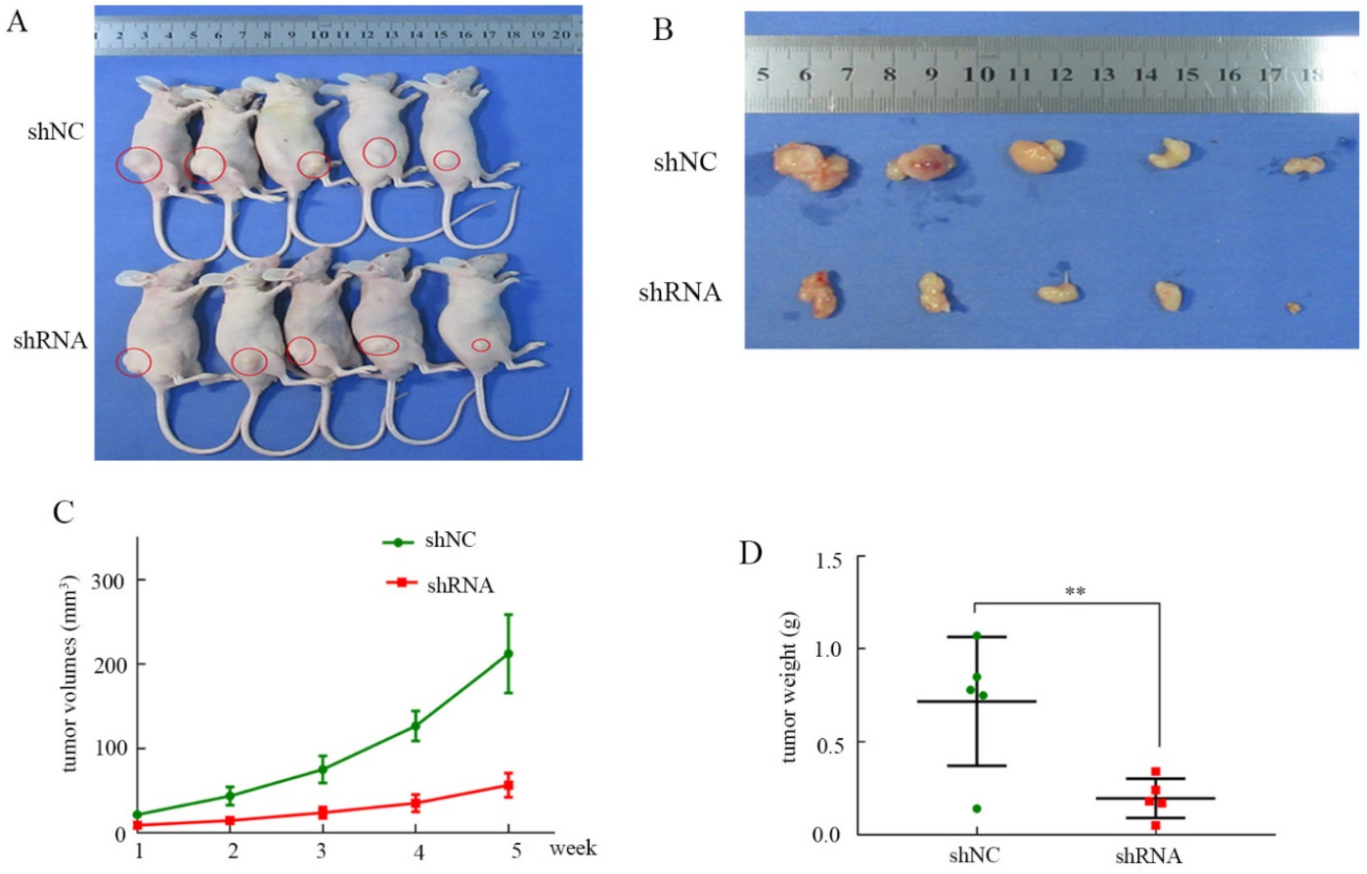
** Tumor growth in node mice. (A)** Tumor growth in node mice receiving BGC-823. **(B)** Tumor shape of shRNA and shNC groups after disection. **(C)** The tumor growth curve in shRNA and shNC groups. **(D)** Tumor weights in shNC and shRNA groups.

To evaluate the influence of *RNF114 in vivo*, a xenograft tumor mouse model was constructed. As shown in Figure 8A-B, knockdown of *RNF114* inhibited tumor growth in the nude mice. The tumor volumes in the shRNA group were significantly lower than those in the shNC group (Figure 8C). Similarly, we clearly showed that the tumor weights were higher in the shNC group than in the shRNA group (Figure 8D).

## Discussion

RNF114 was first reported as a novel susceptibility gene for psoriasis, while several studies have demonstrated that RNF114 plays a critical role in tumor development 36,37. Genetic and environmental changes play a decisive role in GC formation. It is well known that geographical environment, diet and lifestyle factors, smoking, and Helicobacter pylori infection are high-risk GC factors 38-40, and changes in these factors can cause somatic gene mutations promoting GC, such as HER2, VEGF, and RAS 41-43. According to existing research, there is a lack of sufficient studies to elucidate the relationship between RNF114 and GC. We first studied and reported the relationship between RNF114 and GC in detail. As shown in Figure 1, using bioinformatics analysis, the expression of RNF114 in GC was significantly higher than that in normal tissues. We verified these results using qRT-PCR and western blotting (Figures 2 and 3). Through cell function experiments, we found that silencing *RNF114* affected the biological behavior of GC cells, such as proliferation and invasion.

As an oncogene, RNF114 can promote ubiquitylation and degradation of various essential tumor suppressor proteins, while the relative mechanisms of RNF114 are still diminished. Han *et al*. 44 verified that RNF114 ubiquitinated p21^WAF1^ and destabilized p27^KIP1^ and P57^KIP2^, whose alterations may be implicated in human tumors. Interestingly, RNF114 may play a crucial assistive role in activating EGR1 ubiquitylation and degradation. The role of EGR1 in GC is reportedly controversial. As described by Zhonghua 45, EGR1 critically activated linc01503, which was significantly elevated and remarkably linked to overall survival in GC. Hai-Ting *et al*. 46 suggested that overexpression of EGR1 enhanced cell proliferation and the cell cycle by mediating transcription of lncRNA-HNF1A-AS1, contributing to GC progression. However, Crawford *et al*. 47 suggested that EGR1 can interact with TBX2 to co-repress EGR1-target gene expression and is a tumor suppressor in breast cancer. The expression of p21 and its downstream molecules involved in apoptosis were inhibited by EGR1 silencing in GC 21. Guangda found that the low expression of EGR1 is a hub gene and is related to the prognosis of HER2-positive GC 48. The results summarized above suggest that the role of EGR1 remains unclear and needs to be studied further in GC.

In a recent study 49,50, nimbolide was shown to recruit and inactivate RNF114 covalent modifications via cysteine-8 (C8) binding in RNF114, thereby protecting tumor suppressors from ubiquitylation. Rodriguez 51 and Liu 52 found that RNF114 is a new partner of A20, which activates nuclear factor-B (NF-B) and modulates the T cell-mediated immune response. Boren 53 also proposed that RNF114 regulates various cell biological functions, such as cellular dsRNA responses, cell cycle progression, NF-κB activity, and T-cell activation. Based on the above RNF114 research, we may have discovered new therapies for GC, such as targeting or immunotherapy drugs. These therapies require further in-depth research before they can be used clinically.

Interestingly, in the Lin study 54, RNF114 may be a potential target gene of miR-338-3p, and enhancing miR-338-3p could distinctly inhibit RNF114 expression with pemphigus. In our study, we predicted that hsa-miR-218-5p may be a potential regulator of RNF114 in GC. The study involving hsa-miR-218-5p specifically targeted the 3′-UTR regions of CDK6, cyclin D1, and BIRC5 to suppress GC development 24,55. In contrast, Xiaolin *et al*. 56 found that SP1 acts as a transcription factor that binds to the DNA transcriptional chain of RNF114 to control transcription levels. A correlation between RNF114 and SP1 was found in our study. Combining Xiaolin's study, we speculate that SP1 controls RNF114 transcription levels by acting as a transcription factor. *RNF114* gene methylation has not been reported; this study is the first to explore the association between *RNF114* methylation and GC patient prognosis. The divergence with *RNF114* methylation was associated with probe corresponding methylated CpG sites. Not only the methylation levels but also the overall prognosis of GC patients differs between the different probes. The methylation status of the *RNF114* promoter was determined by MSP, however, this function of methylation needs further investigation.

Overall, this research suggests that RNF114 may be a pivotal factor in GC. Regrettably, our team did not further elucidate the upstream regulatory mechanism and downstream ubiquitin-binding proteins via experiments. Therefore, further research is needed to elucidate the role of RNF114 and its molecular mechanism in GC.

## Figures and Tables

**Table 1:** Association between the expression of RNF114 and clinicopathological characteristics of patients with GC

Clinicopathological characteristics	n	Low expression	High expression	χ^2^	P-value
Total	123	57	66		
**Gender**				1.665	0.197
Male	88	44	44		
Female	35	13	22		
**Age (y)**				0.109	0.741
<60	71	32	39		
≥60	52	25	27		
**Tumor localization**				12.139	0.000*
Down	92	51	41		
Up	31	6	25		
**Tumor diameter (cm)**				2.928	0.087
<4	61	33	28		
≥4	62	24	38		
**Tumor differentiation**				0.875	0.350
Low	85	37	48		
Middle + High	38	20	18		
**T stage**				8.734	0.003
T1+T2	32	22	10		
T3+T4	91	35	56		
**Lymph node metastasis**				12.032	0.001
no	39	27	12		
yes	84	30	54		
**Cancer nodule number**				3.037	0.104*
no	113	55	58		
yes	10	2	8		
**Vascular invasion**				2.603	0.107
no	77	40	37		
yes	46	17	29		

* Fisher's exact test.

**Table 2:** Univariate and multivariate analysis of the association between prognosis, clinicopathological characteristics, and RNF114 expression in GC

Variable	Univariate analysis	Multivariable analysis	
	P > |z|	P > |z|	HR (95%CI)
**RNF114 expression**	0.000	0.015	1.115-2.817 (1.772)
Low (n = 57) vs high (n = 66)			
**Gender**	0.758	NA	NA
Male (n = 88) vs female (n = 35)			
**Age (y)**	0.668	NA	NA
<60 (n = 71) vs ≥60 (n = 52)			
**Tumor differentiation**	0.283	NA	NA
Low (n = 91) vs middle + high (n =32)			
**Tumor localization**	0.008	0.938	0.608-1.583 (0.981)
Up (n = 31) vs down (n = 92)			
**Tumor diameter (cm)**	0.000	0.203	0.860-2.034 (1.323)
<4 (n = 61) vs ≥4 (n = 62)			
**T stage**	0.000	0.000	4.807-22.638 (10.432)
T1+T2 (n = 32) vs T3+T4 (n = 91)			
**N stage**	0.000	0.000	2.612-7.769 (4.504)
No (n = 39) vs Yes (n = 84)			
**Cancer nodule number**	0.000	0.015	1.192-5.133 (2.437)
No (n = 113) vs Yes (n = 10)			
**Vascular invasion**	0.000	0.091	0.939-2.318 (1.476)
No (n = 77) vs Yes (n = 46)			

NA: not analyzed.
